# The Prognostic Impact of the Metastatic Lymph Nodes Ratio in Colorectal Cancer

**DOI:** 10.3389/fonc.2018.00628

**Published:** 2018-12-18

**Authors:** Chi-Hao Zhang, Yan-Yan Li, Qing-Wei Zhang, Alberto Biondi, Valeria Fico, Roberto Persiani, Xiao-Chun Ni, Meng Luo

**Affiliations:** ^1^Department of General Surgery, Shanghai Ninth People's Hospital, School of Medicine, Shanghai Jiao Tong University, Shanghai, China; ^2^Department of Radiation Oncology, Shanghai Ninth People's Hospital, School of Medicine, Shanghai Jiao Tong University, Shanghai, China; ^3^Division of Gastroenterology and Hepatology, Key Laboratory of Gastroenterology and Hepatology, Ministry of Health, Renji Hospital, School of Medicine, Shanghai Jiao Tong University, Shanghai Institute of Digestive Disease, Shanghai, China; ^4^Dipartimento Scienze Gastroenterologiche ed Endocrino-Metaboliche, Fondazione Policlinico Universitario A. Gemelli IRCCS, Università Cattolica del Sacro Cuore, Rome, Italy

**Keywords:** Colorectal cancer, lymph node ratio, prognosis, overall survival, SEER database

## Abstract

**Background:** This study was designed to validate the prognostic significance of the ratio of positive to examined lymph nodes (LNR) in patients with colorectal cancer.

**Methods:** 218,314 patients from the SEER database and 1,811 patients from the three independent multicenter were included in this study. The patients were divided into 5 groups on a basis of previous published LNR: LNR0, patients with no metastatic lymph nodes; LNR1, patients with the LNR between 0.1 and 0.17; LNR2, patients with the LNR between 0.18 and 0.41; LNR3, patients with the LNR between 0.42 and 0.69; LNR4, patients with the LNR >0.7. The 5-year OS and CSS rate were estimated using Kaplan-Meier method and the survival difference was tested using log-rank test. Multivariate Cox analysis was used to further assess the influence of the LNR on patients' outcome.

**Results:** The 5-year OS rate of patients within LNR0 to LNR4 group was 71.2, 55.8, 39.3, 22.6, and 14.6%, respectively (*p* < 0.001) in the SEER database. While the 5-year OS rate of those with LNR0 to LNR4 was 75.2, 66.1, 48.0, 34.0, and 17.7%, respectively (*p* < 0.001) in the international multicenter cohort. In the multivariate analysis, LNR was demonstrated to be a strong prognostic factor in patients with < 12 and ≥12 metastatic lymph nodes. Furthermore, the LNR had a similar impact on the patients' prognosis in colon cancer and rectal cancer.

**Conclusion:** The LNR allowed better prognostic stratification than the positive node (pN) in patients with colorectal cancer and the cut-off values were well validated.

## Introduction

Colorectal cancer (CRC) is the fourth most common malignancy in America. The estimated number of cancer-related deaths in 2015 reached 49,190 in 2015 (1). Currently, the TNM staging system is the most frequently used tool in clinical practice to stage the tumor in CRC patients and to evaluate their long-term survival outcome. Patients were divided into groups with different prognosis according to the depth of invasion of primary tumors, the number of positive lymph nodes (LNs), and the presence of distant metastatic sites. LN status is defined by the number of metastatic LNs harvested. Prognosis worsens when the number of metastatic LNs increases: pN0 (no nodes involved), pN1 (involved nodes, ≤ 3) and pN2 (involved nodes, >3). In addition, the 5-year survival rate of the patients with stage II colon cancer is approximately 80%. While the rate drops to 50% in stage III colon cancer patients with LN metastasis. Therefore, the number of metastatic LNs has been established as an important prognostic determinant in patients with CRC.

To ensure accurate staging, many studies were carried out in patients with metastatic CRC to explore the correct number of LNs that should be examined. However, the results varied largely among these studies, ranging from as few as 6 to as many as 40, because the number of examined LNs depends on the specialty of surgeons, Dukes' stage, specimen length, tumor size, individual immune response and skill level of pathology technicians (2–5). According to recent publications, ratio-based LN staging emerged as a substitute for the positive LN (pN) count. In fact, its impact on survival has been proven in some malignant tumors including pancreatic, gastric, esophageal, and bladder cancer (6–8). A similar result was also observed among individuals with CRC (9–11). One of the biggest features of LNR is that its survival predictive capacity seems less dependent on the number of resected nodes. Because of this, the LNR classification may reduce the risk of over- or under-staging in comparison with traditional pN staging. However, the reported cut-off values of LNR vary and no consensus has been reached (12–15). Most of those studies used mean value, quartiles or an arbitrary classification to calculate ratio-based LN values, and only three of them used statistical methods.

In a previous study, Rosenberg et al. divided 3,026 patients into five groups according to the cut-off values of LNR, which were calculated using the classification and regression trees method (16). The ratios were 0, 0–0.17, 0.18–0.41, 0.42–0.69, and >0.7. They demonstrated that these values had the highest survival impact and stressed its function in the prediction of the survival of CRC patients as an independent prognostic factor. The values were also examined by Rosenberg and his colleagues in another large population from a single medical center, and the results showed the strong prognostic value of LNR (17). To further validate the discriminatory performance of these previously published cut-off values, patients from the SEER database and three independent cross-international medical centers were included in this study and were analyzed.

## Materials and Methods

### Surveillance, Epidemiology, and End Results Database

**Figure 1:**
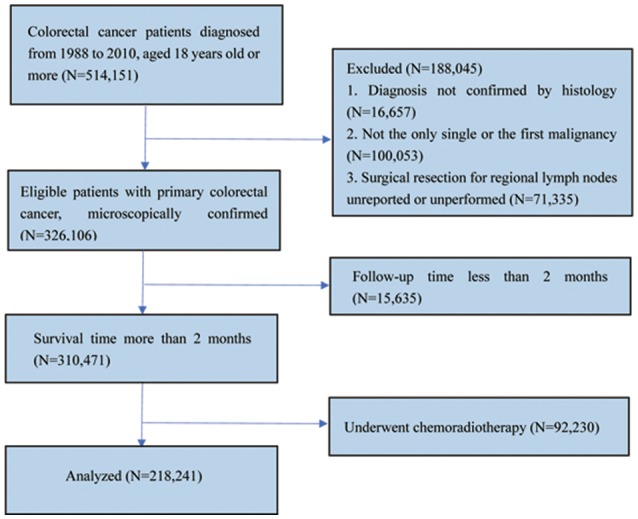
Flow chart of patients' cohort definition from the SEER database.

Data of patients with CRCs from Surveillance, Epidemiology and End Results (SEER) database was searched and collected through queries using the latest version of the SEER 18 Regs Research Data (1973–2014), released in April 2017 with the SEER^*^Stat 8.3.5 software. The inclusion criteria for selected patients were as follows: (1) Patients aged 18 years or older who were diagnosed between 1988 and 2010; (2) patients with CRC diagnosed as the only primary cancer without multiple primary cancer elsewhere; (3) patients with cancer diagnosed microscopically, in whom surgery for primary cancer and regional LN resection had been performed with pathological examination of at least one LN; (4) patients' whose CRC was defined using the International Classification of Diseases for Oncology third edition (ICD-O-3) codes (8,010–8,231 and 8,255–8,576); and (5) patients who were followed up for at least 2 months were included in the study. Patients who received chemoradiotherapy at any time or patients' whose CRC was not the only primary carcinoma were excluded (Figure 1).

For SEER database, cancer-specific survival (CSS) was the primary outcome, while overall survival (OS) and CSS considering competing death as due to non-CRC death were the secondary outcomes. Survival time was defined as the time from diagnosis to the date of death or last contact or November 2016.

Since the SEER database includes public-use data, no institutional review was required. We were allowed to access the SEER database for research purposes only using the private SEER ID (Zhang).

### International Multicentre Cohort

An independent international multicentre cohort from three medical centers was used as another validation group using the same inclusion and exclusion criteria (Supplementary Figure 1). Patients with histopathologically confirmed CRC who underwent colectomy from February 2009 to March 2013 in Shanghai Ninth People's Hospital of Shanghai Jiao Tong University and Renji Hospital of Shanghai Jiao Tong University, and from January 2004 to April 2017 in Catholic University of Rome were selected. The patients were followed up every 6 months until death or until the end of the study (30 March 2018) in the two Chinese hospitals, except for those lost to follow-up. For patients in the Catholic University of Rome, they were followed up until death or until end of the study (30 April 2018) except for those lost to follow-up according to the European Society of Medical Oncology guidelines.

The study protocol for the international multicentre cohort was approved by the Institutional Review Board of the Shanghai Ninth People's Hospital, Renji Hospital, and the Catholic University of Rome.

### Statistical Analysis

Descriptive statistics were used to examine and report the patient demographic characteristics. All quantitative variables were represented as means with 95% confidence intervals (CI). χ^2^-test was used to compare the differences of variable distribution between groups. The end point of our study was OS and CSS. Survival curves were plotted using the Kaplan-Meier method, and the difference between curves was compared using the log rank test. Multivariate analyses were performed with Cox proportional hazards regression model to evaluate the potential covariates in relation to 5-year OS and CSS. In addition, we conducted subgroup analyses according to the tumor location (colon vs. rectum) and the number of LNs examined with a prespecified cut-off value of 12. The Akaike information criterion (AIC) was used to compare the quality of different statistical models. The smaller value means a better fit and model for predicting survival outcome. Besides, the cumulative probability of CRC-specific death was calculated and multivariate regression modeling of sub-distribution functions in competing risks were performed for sensitivity analysis of our findings using the cmprsk package. All statistical analyses were performed using SPSS version 13.0 (SPSS, Chicago, IL, United States) and R software for Windows (version R-3.4.3, the R Foundation for statistical computing). All statistical comparisons were two-sided. A *P* < 0.05 was regarded as a threshold of statistical significance.

## Results

### Patient Characteristics From SEER Database and the International Multicentre Cohort

According to the inclusion and exclusion criteria, a total of 218,341 patients with CRC were finally selected from the SEER database, including 103,614 men and 114,727 women. The mean age of the participants was 68.1 years. The mean number of retrieved LNs was 14.2. The mean numbers of positive and negative LNs were 1.6 and 12.7, respectively. According to the 7th American Joint Committee on Cancer (AJCC) staging system, the number of patients categorized as pN1 was 50,753, while the number of those categorized as pN2 was 32,306. The rest of the patients had no node involvement.

In the multicentre data which included 1,811 patients, 1,330 were diagnosed with colon cancer and 464 had rectal cancer. The mean number of retrieved LNs was 10.5. The mean numbers of positive and negative LNs were 1.3 and 9.2, respectively. Their mean follow-up time was 49.6 months. About 462 patients were categorized as pN1, and 238 patients were categorized as pN2. More detailed information can be seen in Table 1.

**Table 1:** Characteristics of patients with colorectal cancer from SEER database and international multicenter cohort.

**Characteristics**	**Number (%)**		***P-*value**
	**SEER**	**International multicenter**	
**SEX**			
Female	114,727(52.5%)	783(43.3%)	<0.001
Male	103,614(47.5%)	1,028(56.7%)	<0.001
**AGE**			
Mean(95%Cl)	68.1 ± 13.5	67.1 ± 11.5	
< 60	57,819(26.5%)	475(26.3%)	0.687
≥ 60	160,522(73.5%)	1,336(73.7%)	0.687
**TUMOR SIZE (CM)**			
Mean(95%Cl)	4.7 ± 2.7	4.9 ± 2.2	<0.001
≤2	23,232(10.6%)	114(6.3%)	<0.001
≤3	32,039(14.7%)	262(14.5%)	<0.001
≤5	76,659(35.1%)	794(43.8%)	<0.001
>5	63,165(28.9%)	622(34.3%)	<0.001
NA	23,246(10.6%)	9(1.0%)	<0.001
**TUMOR SITE**			
Left side	72,103(33.0%)	754(41.6%)	<0.001
Right side	108,772(49.8%)	576(31.8%)	<0.001
Rectum	34,597(15.8%)	464(25.6%)	<0.001
NA	2,869(1.3%)	17(0.9%)	<0.001
**HISTOLOGY**			
Adenocarcinoma	192,081(88.0%)	1,630(90.0%)	<0.001
Mucinous adenocarcinoma	22,968(10.5%)	178(9.8%)	<0.001
Signet ring cell carcinoma	2,001(0.9%)	3(0.2%)	<0.001
Others	1,291(0.6%)	0(0%)	<0.001
**DIFFERENTIATION**			
Well/Moderately differentiated	167,896(76.9%)	494(27.3%)	<0.001
Poorly or undifferentiated	40,416(18.5%)	1285(71.0%)	<0.001
NA	10,029(4.6%)	32(1.8%)	<0.001
**7th T STAGE**			
T1	31,045(14.2%)	113(6.2%)	<0.001
T2	35,033(16.0%)	219(12.1%)	<0.001
T3	124,060(56.8%)	1,211(66.9%)	<0.001
T4	28,203(12.9%)	268(14.8%)	<0.001
**7th M STAGE**			
M0	20,2131(92.6%)	1,678(92.7%)	0.951
M1	16,210(7.4%)	133(7.3%)	0.951
Total no. of nodes retrieved [Mean(95%Cl)]	14.2 ± 9.6	10.5 ± 6.4	<0.001
No. of negative nodes (nN) [Mean(95%Cl)]	12.7 ± 9.5	9.2 ± 6.3	<0.001
No. of positive nodes (pN) [Mean(95%Cl)]	1.6 ± 3.4	1.3 ± 2.5	0.992
**7th N STAGE (7th pN)**			
N0	135,282(62.0%)	1111(61.3%)	0.008
N1a	24,912(11.4%)	212(11.7%)	0.008
N1b	25,841(11.8%)	250(13.8%)	0.008
N2a	17,399(8.0%)	145(8.0%)	0.008
N2b	14,907(6.8%)	93(5.1%)	0.008
Follow-up(months) Mean(95%Cl)	82.4 ± 63.0	49.6 ± 30.3	<0.001

*CI, confidence interval*.

**Figure 2:**
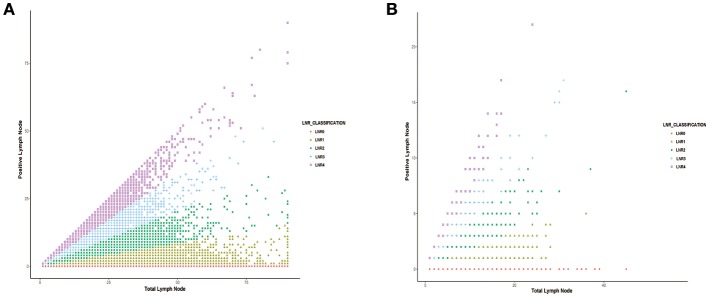
The correlation between the number of positive lymph nodes and the number of total lymph node harvested in **(A)** SEER database and **(B)** international multicenter cohort.

As shown in Figure 2, the association between positive LNs and total LNs was plotted based on the data from SEER database (Figure 2A) and multicentre cohort (Figure 2B). The absolute number of pN increased as the examined LNs increase.

### Distribution of Clinical and Histopathologic Characteristics Based on Lymph Node Ratios

In the SEER database, 135,282 patients were classified as LNR0 with 5-year OS and CSS of 71.2 and 84.7%, respectively. A total of 35,047 patients was classified as LNR1 with 5-year OS and CSS of 55.8 and 64.2%, respectively. A total of 25,362 patients was classified as LNR2 with 5-year OS and CSS of 39.3 and 45.9%, respectively. A total of 13,046 patients was classified as LNR3 with 5-year OS and CSS of 22.6 and 31.2%, respectively. A total of 9,605 patients was classified as LNR4 with 5-year OS and CSS of 14.6 and 17.7%, respectively (Table 2).

**Table 2:** Association between clinicopathologic characteristics and lymph node ratio (LNR) of patients with colorectal cancer in SEER database.

	**Positive Lymph Node Ratios**												
	**Total**		**0**		**0.01–0.17**		**0.18–0.41**		**0.42–0.69**		**≥0.70**		***P***
	***N***	**Percent**	***N***	**Percent**	***N***	**Percent**	***N***	**Percent**	***N***	**Percent**	***N***	**Percent**	
pT													<0.001
T1	31,045	14.2	28,398	21.0	1,538	4.4	697	2.7	252	1.9	160	1.7	
T2	35,033	16.0	28,781	21.3	3,442	9.8	1,818	7.2	666	5.1	326	3.4	
T3	124,060	56.8	66,705	49.3	24,559	70.1	17,833	70.3	8,980	68.8	5,983	62.3	
T4	28,203	12.9	11,398	8.4	5,507	15.7	5,014	19.8	3,148	24.1	3,136	32.6	
pN													<0.001
N0	135,282	62.0	135,282	100	0	0	0	0	0	0	0	0	
N1a	24,912	11.4	0	0	20,908	59.7	2,917	11.5	552	4.2	535	5.6	
N1b	25,841	11.8	0	0	12,031	34.3	10,308	40.6	2,455	18.8	1,047	10.9	
N2a	17,399	8.0	0	0	1,929	5.5	9,090	35.8	4,333	33.2	2,047	21.3	
N2b	14,907	6.8	0	0	178	0.5	3,047	12.0	5,706	43.7	5,976	62.2	
EN	14.2 ± 9.6		13.43 ± 9.46		19.07 ± 10.57		13.87 ± 8.18		12.48 ± 7.76		11.49 ± 8.66		<0.001
PN	1.6 ± 3.4		0		1.67 ± 1.07		3.88 ± 2.48		6.75 ± 4.36		9.88 ± 7.59		<0.001
NN	12.7 ± 9.5		13.43 ± 9.46		17.41 ± 10.08		9.99 ± 6.06		5.73 ± 3.74		1.60 ± 1.86		<0.001
M													<0.001
M0	202,131	92.6	132,340	97.8	31,604	90.2	21,294	84.0	10,125	77.6	6,768	70.5	
M1	16,210	7.4	2,942	2.2	3,442	9.8	4,068	16.0	2,921	22.4	2,837	29.5	
OS	58.3%		71.2%		55.8%		39.3%		22.6%		14.6%		<0.001
CSS	68.9%		84.7%		64.2%		45.9%		31.2%		17.7%		<0.001

*EN, examined nodes; PN, positive nodes; NN, negative nodes; OS, overall survival; CSS, cancer-specific survival*.

In our own collected data, 1,112 patients had no positive LNs, and the 5-year OS was 75.2%. A total of 232 patients was classified as LNR1 with a 5-year OS of 66.1%. A total of 246 patients was classified as LNR2 with a 5-year OS of 48.0%. A total of 132 patients was classified as LNR3 with a 5-year OS of 34.0%. A total of 89 patients was classified as LNR4 with a 5-year OS of 15.0% (Supplementary Table 1).

### Impact of the pN on OS and CSS in Patients With Colorectal Cancer

We analyzed the effects of pN on patients' survival in SEER database using the multivariate Cox model. As shown in Table 3, the pN was a strong prognostic factor in predicting patients' outcome. In addition, gender (*P* < 0.001), race (*P* < 0.001), age (*P* < 0.001), tumor location (*P* < 0.001), pathological grade (*P* < 0.001), histological type (*P* < 0.001), T stage (*P* < 0.001), and M stage (*P* < 0.001), as well as tumor size (*P* < 0.001) were correlated with the rate of CRC patients' OS and CSS. Similarly, our own data also demonstrated that pN was an independent factor in the prediction of survival of CRC patients (Supplementary Table 2, *P* < 0.001).

**Table 3:** Multivariate analysis of pN and clinicopathologic characteristics for overall survival (OS) and cancer-specific survival (CSS) in SEER colorectal cancer patients.

**Characteristics**	**Overall survival**		**Cancer-specific survival**	
	**HR**	***p***	**HR**	***p***
**SEX**				
Male vs. Female^*^	1.09 (1.07–1.10)	<0.001	1.08 (1.06–1.10)	<0.001
**RACE**				
White	Reference			
Black	1.11 (1.09–1.13)	<0.001	1.23 (1.20–1.26)	<0.001
Other	0.78 (0.76–0.80)	<0.001	0.83 (0.81–0.86)	<0.001
**AGE LEVEL**				
≥60 vs. <60^*^	2.71 (2.66–2.75)	<0.001	1.65 (1.61–1.68)	<0.001
**LOCATION**				
Right side	Reference			
Left side	0.94 (0.93–0.96)	<0.001	1.01 (0.99–1.03)	0.499
Rectum	1.11 (1.09–1.13)	<0.001	1.29 (1.26–1.32)	<0.001
**GRADE**				
High grade vs. Low grade^*^	1.15(1.13–1.16)	<0.001	1.23 (1.21–1.26)	<0.001
**HISTOLOGY**				
Adenocarcinoma	Reference			
Mucinous adenocarcinoma	1.01 (0.99–1.03)	0.165	1.02 (0.99–1.04)	0.242
Signet ring cell carcinoma	1.39 (1.31–1.47)	<0.001	1.37 (1.28–1.46)	<0.001
Others	1.23 (1.14–1.34)	<0.001	1.41 (1.27–1.55)	<0.001
**T**				
T1	Reference			
T2	1.19 (1.15–1.23)	<0.001	1.52(1.42–1.62)	<0.001
T3	1.54 (1.49–1.58)	<0.001	2.90 (2.73–3.08)	<0.001
T4	2.19 (2.12–2.26)	<0.001	4.87 (4.57–5.18)	<0.001
**N**				
N0	Reference			
N1	1.31 (1.29–1.33)	<0.001	2.06 (2.02–2.10)	<0.001
N2	1.99 (1.95–2.02)	<0.001	3.41 (3.33–3.49)	<0.001
**M**				
M1 vs. M0^*^	3.22 (3.15–3.28)	<0.001	3.56 (3.48–3.64)	<0.001
**SIZE LEVEL**				
≤2	Reference			
≤3	1.07 (1.04–1.10)	<0.001	1.06 (1.02–1.11)	<0.001
≤5	1.10 (1.08–1.13)	<0.001	1.09 (1.05–1.14)	<0.001
>5	1.13 (1.10–1.16)	<0.001	1.12 (1.08–1.17)	<0.001

*HR, hazard ratio; CI, confidence interval*;

* *Reference category*.

### Impact of the LNR on OS and CSS in Patients With Colorectal Cancer

**Figure 3:**
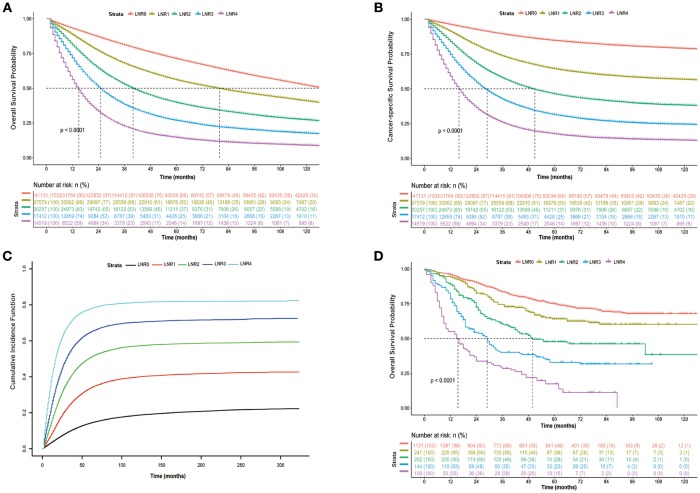
Kaplan-Meier cumulative survival curves of CRC patients stratified by lymph node ratio (LNR 0–4). **(A)** Kaplan-Meier analysis of overall survival (OS) of CRC patients from SEER database. **(B)** Kaplan-Meier analysis of cancer-specific survival (CSS) of CRC patients from SEER database. **(C)** Cumulative probability of CRC-specific death in competing risks. **(D)** Kaplan-Meier analysis of overall survival (OS) of CRC patients from international multicenter cohort.

As shown in Figure 3, patients in the high LNR group had poorer OS (Figure 3A, *P* < 0.001), poorer CSS (Figure 3B, *P* < 0.001), and higher cumulative probability of cancer-specific death (Figure 3C, *P* < 0.001) than those in relatively low LNR group. In multivariate Cox analysis of data from SEER database, we found that the LNR was associated with poor OS and CSS in CRC patients. Meanwhile, gender (*P* < 0.001), race (*P* < 0.001), age (*P* < 0.001), tumor location (*P* < 0.001), pathological grade (*P* < 0.001), histological type (*P* < 0.001), T (*P* < 0.001), and M stage (*P* < 0.001) as well as tumor size (*P* < 0.001) were considered as independent prognostic factors. We then divided the patients into rectal cancer group and colon rectal group according to the position of the primary tumor. The multivariate analysis also showed that the LNR had a great influence on patients' survival (Supplementary Figures 2, 3, *P* < 0.001). The adequate resected LNs count was taken as a guarantee for accurate tumor staging. Therefore, we further analyzed the impact of LNR on OS and CSS in both patients with < 12 and ≥12 resected nodes. The results were similar to the findings of the above analyses (Supplementary Figures 4, 5, *P* < 0.001).

In the analysis of our own data, the results showed that the patient's OS decreased when the LNR increased (Figure 3D, *P* < 0.001). Multivariate analysis was also performed and showed that the LNR still had a great influence on patients' outcome (Supplementary Table 3, *P* < 0.001). In addition, we also calculated the AIC of the models with pN or LNR and found that the AIC was smaller in the model with LNR (2,431,814 and 1,251,010 for OS and CSS, respectively) compared with the model with pN (2,434,079 and 1,253,289 for OS and CSS, respectively), indicating that the LNR may be a better prognostic factor in comparison to pN.

### Five-Year OS and CSS in LNR Groups

We calculated the 5-year OS and CSS for all patients and pN1 and pN2 patients. The results showed that patients' survival shortened as the LNR increased. When we examined the OS and CSS of patients categorized as pN1, we found that the 5-year OS and CSS rates was 62.1 and 64.8%, respectively, in patients with LNR smaller than 0.17. While the OS and CSS rates dropped to 23.3 and 29.2% when the patients had a LNR >0.7. Furthermore, within the pN2 group, it is not difficult to notice a huge difference in 5-year OS and CSS rate between patients with a LNR smaller than 0.17 and those with a LNR >0.7 (OS: 49.3 vs. 12.8%; CSS: 55.1 vs. 15.3%). In our data, only seven patients within the pN2 group who had a LNR smaller than 0.17. We suppose that the OS rate may be affected by the sample size and is very likely to be inaccurate. However, the survival rate also decreased with increasing LNR (Table 4).

**Table 4:** Five-Year Survival Rates for patients with colorectal cancer stratified by LN radio and pN category in SEER database and International multicenter.

**Groups**	**Overall(%)**	**LNR1(%)**	**LNR2(%)**	**LNR3(%)**	**LNR4(%)**
**5-YEAR SURVIVAL RATES BY LN RATIO (SEER)**					
**N1 Patients**					
OS	49.0	62.1	41.3	31.0	23.3
CSS	57.2	64.8	49.2	37.7	29.2
**N2 Patients**					
OS	27.1	49.3	37.0	25.2	12.8
CSS	31.2	55.1	42.2	29.2	15.3
**5-YEAR SURVIVAL RATES BY LN RATIO (INTERNATIONAL MULTICENTER)**					
**N1 Patients**					
OS	53.2	67.8	44.7	34.7	26.0
**N2 Patients**					
OS	32.8	—	58.0	33.8	9.6

*OS, overall survival; CSS, cancer-specific survival; LNR, lymph node radio*.

## Discussion

In the present study, we presented a population-based analysis of 240,898 patients in the SEER database and international multicentre analysis of 1,878 patients in three medical centers. Our analysis provided evidence that the cut-off values of LNR proposed by Rosenberg et al. were well validated and led to significant survival stratification. More importantly, compared with the TNM staging system, the novel staging system had stronger prognostic concordance in the present cohort. We also demonstrated that this LNR classification had a good performance in patients who underwent resection of <12 and ≥12 LNs. Furthermore, the LNR was found to have a similar impact on the prognosis of patients with colon and rectal cancer. To our knowledge, this is the largest multicentre study to assess the value of LNR in the prediction of CRC patients' survival.

It is worth mentioning that although most of the characteristics of patients between two cohorts were statistically significant, this result may be partly attributed to the large sample size of the data from the SEER database. However, the confounding factors in both cohorts were adjusted, to some extent, in the multivariate Cox regression model. Moreover, based on the analysis, the two cohorts had similar results, further suggesting the strong prognostic role of LNR in patients with metastatic CRC.

It is well established that positive LN count has a strong impact on the prognosis of CRC patients (18, 19). Hence, a thorough LN evaluation in CRC will ensure accurate patient staging, thus offering important information regarding the prediction of patients' long-term prognosis, and allow more reasonable adjuvant chemoradiotherapy. However, the prognostic value of pN stage is, to some extent, limited due to its relationship with the resected LNs. Several studies demonstrated that the number of metastatic nodes has a positive correlation with the total LN harvested, which was also confirmed by our study, suggesting that the risk of cancers with inadequate LNs may be underestimated because of sampling error (13, 20, 21).

As a result, the AJCC and other professional organizations recommended that a minimum of 12 nodes was required for accurate tumor staging (22, 23). However, some pointed out that this number had no particular biological significance; on the contrary, it is likely a consequence of a statistical probability distribution, suggesting that once >12 nodes have been harvested, the likelihood of missing the remaining positive mesenteric node becomes very small (24). Many factors may be associated with the retrieval of the ultimate number of LNs, including specialty of surgeon, tumor location, stage of disease, and preoperative radiotherapy.

In an attempt to overcome these limitations, LNR, as a significant prognostic factor, has been put forward and reported in CRC by several studies (24–26). Nearly all studies reported that LNR had an excellent performance in predicting patients' survival outcome (27). However, no consensus was achieved on the best cut-off value. On the other hand, the methods used to calculate cut-off values varied across different studies, such as the median value, quartiles of distribution or arbitrary values (28–30). Only three of these groups applied statistical approaches to stratify LNR for the discrimination of patients' outcome. In the first study, the cut-off value was based on SEER data from 26,186 patients and was identified 0.4 as the optimal categorization of the LNR (20). The second study was carried out on patients with rectal cancer, and colon cancer was not considered. In another study, the cut-off points were 0.17, 0.41 and 0.69 with high prognostic impact in CRC. It is not difficult to note that one of the values mentioned above was close to 0.4; on this basis, the researchers further optimize the stratification with another two values. Meanwhile, both rectal cancer and colon cancer were included in this study. That is why we finally chose these values to be validated in our current work.

Our study found that LNR staging is superior to traditional TNM classification in tumor staging. This conclusion is based on the fact that the difference of 5-year OS rate between LNR1 and LNR4 in patients with pN1 is 33.8% (*P* < 0.001). Further analysis has shown that 5-year OS and CSS rate of patients in pN1 with LNR4 was much poorer than those in pN2 with LNR1. Nevertheless, this survival difference cannot be effectively reflected by TNM staging system. On the contrary, the survival rates were very close when patients were in the same LNR group. We also calculated the AIC for the model with LNR or pN incorporated. AIC was usually used to evaluate the quality of the model. A smaller value indicates a better model (31). In our study, we found that AIC in the model with LNR was smaller than that in the model with pN, suggesting that LNR was a superior prognostic factor in comparison to pN. Therefore, using the LNR as a staging system for CRC could decrease the possibility of stage migration compared with the traditional AJCC classification system.

It is well-known that the removal of a higher number of LNs (>12) guarantees the accuracy for CRC staging. In other words, pN staging may not be able to accurately predict CRC patients' prognosis when <12 LNs are resected. As a candidate, the role of LNR in predicting CRC patients' survival outcome when a lesser number of LNs are harvested remains controversial. Berger et al. reported that the influence of the LNR on CRC patients' survival was not significant when <10 LNs were dissected (15). In that case, they suggested that the total number of positive LNs was a more important factor in prognostic prediction. In contrast, Wang et al. found that the ability of LNR in prognostic prediction was not dependent on the number of LNs retrieved (26). For patients with fewer than 10 LNs, the LNR remained the most important prognostic factor. Therefore, in order to assess the real prognostic ability of LNR in CRC patients with lower or higher number of harvested LNs, we performed a subgroup analysis based on the number of LN harvested (<12 vs. ≥12). In our study conducted in 79,012 patients with examined LNs <12, the 5-year OS and CSS rates of the patients in the high LNR category was significantly worse than those of patients in the low LNR category. We can see the similar trend in cases with >12 LNs harvested. Multivariate analysis was performed to adjust the influence of other potential factors, and results have shown that the LNR still had a good prognostic capability when the patients had <12 LNs dissected. Thus, the determination of the LNR may to some extent compensate for an inadequate number of LNs harvested in CRC surgery. Nevertheless, this finding should be carefully viewed. An adequate number of LNs harvested still plays an important role in the accurate assessment of patients' prognosis. Thus, more large-scale studies are still needed to further confirm the value of LNR.

With regard to tumor localization, a resected colon specimen was found to contain more LNs than a resected rectal specimen. On the other hand, patients with rectal cancer frequently undergo preoperative chemoradiotherapy compared with those with colon cancer. Several studies reported that the use of preoperative CRT was highly correlated with the smaller number of LN harvested (32–34). Therefore, inadequate LN removal may occur more easily in patients with rectal cancer and result in overstaging or understaging when using pN category. To evaluate whether LNR can be affected by the total LN yield, subgroup analyses were performed based on the tumor location. Results showed that LNR had similar impact on the survival outcome of colon and rectal cancer patients.

There were some limitations in our study. Firstly, this study was a retrospective study, and might not guarantee the completeness of the data. For example, although the average follow-up time of patients from the international multicentre cohort was 9.55 years and was considered acceptable, CSS could not be calculated because data on the cause of death was not available. An additional limitation was the absence of the detailed information of adjuvant therapy in the SEER database. With this, it would be impossible to evaluate or adjust the treatment effect. However, patients who underwent any chemoradiotherapy were excluded. This could reduce the bias to some extent. Moreover, the sample size of the multicentre cohort was relatively small compared with the SEER database. Hence, our results should be externally validated in another large multicentre-based cohort or population-based register.

## Conclusion

In summary, the cut-off values of LNR by Rosenberg et al. offer better prognostic stratification for CRC in comparison with the number of positive LNs. The staging system that took LNR into account may offer more survival information to patients and play a role in treatment decisions.

## Ethics Statement

The study was approved by the Ethics Committee of Shanghai Ninth People's Hospital, Renji Hospital and the Catholic University of Rome. As this is a retrospective study in nature, the informed consent was not required in this study.

## Author Contributions

AB, VF, and RP contributed to data acquisition. C-HZ and Y-YL performed the statistical analysis and prepared the manuscript. Q-WZ drafted this manuscript. X-CN and ML supervised the study. All authors read and approved the final manuscript.

### Conflict of Interest Statement

The authors declare that the research was conducted in the absence of any commercial or financial relationships that could be construed as a potential conflict of interest.

## Abbreviations

LNR: ratio of positive to examined lymph nodes
pN: positive node
CRC: Colorectal cancer
LN: Lymph node
SEER database, Surveillance, Epidemiology: and End Results database
CSS: cancer-specific survival
OS: overall survival
CI: confidence intervals
AIC: Akaike information criterion.
