# Supplementary material for: The Prognostic Impact of the Metastatic Lymph Nodes Ratio in Colorectal Cancer
Source: Front Oncol. 2018 Dec 18;8:628. doi: 10.3389/fonc.2018.00628 (PMC6305371; doi:10.3389/fonc.2018.00628)
Supplement: Supplementary file 1 [file Data_Sheet_1.docx]

Supplementary Material

The prognostic impact of the metastatic lymph nodes ratio in colorectal cancer

Chi-Hao Zhang^1. *^, Yan-Yan Li^2. *^, Qing-Wei Zhang^3. *^, Alberto Biondi^4^, Valeria Fico^4^, Roberto Persiani^4^, Xiao-Chun Ni^1. #^, Meng Luo^1. #^

*Chi-Hao Zhang, Yan-Yan Li and Qing-Wei Zhang contributed equally to this work.

#Xiao-Chun Ni and Meng Luo are both corresponding authors of this paper.

Correspondences: Professor Meng Luo, E-mail: [luosh9hospital@sina.com](mailto:luosh9hospital@sina.com);

Xiao-Chun Ni, Email: nixiaochun1981@aliyun.com.

## Supplementary Tables:

**Supplementary Table 1. Association between clinicopathologic characteristics and lymph node ratio (LNR) of patients with colorectal cancer in international multicenter cohort.**

|  | | | Positive Lymph Node Ratios | | | | | | | | | | | | | | | | |  |
| --- | --- | --- | --- | --- | --- | --- | --- | --- | --- | --- | --- | --- | --- | --- | --- | --- | --- | --- | --- | --- |
|  | Total | | |  | 0 | |  | 0.01 – 0.17 | |  | 0.18 – 0.41 | |  | 0.42 – 0.69 | |  | > =0.70 | | *P* |  |
|  | N | Percent | |  | N | Percent |  | N | Percent |  | N | Percent |  | N | Percent |  | N | Percent |  |  |
| pT |  |  | |  |  |  |  |  |  |  |  |  |  |  |  |  |  |  | < 0.001 |  |
| T1 | 113 | 6.2 | |  | 109 | 9.8 |  | 2 | 0.9 |  | 2 | 0.8 |  | 0 | 0 |  | 0 | 0 |  |  |
| T2 | 219 | 12.1 | |  | 193 | 17.4 |  | 14 | 6.0 |  | 5 | 2.0 |  | 4 | 3.0 |  | 3 | 3.4 |  |  |
| T3 | 1211 | 66.9 | |  | 702 | 63.1 |  | 164 | 70.7 |  | 178 | 72.4 |  | 99 | 75.0 |  | 68 | 76.4 |  |  |
| T4 | 268 | 14.8 | |  | 108 | 9.7 |  | 52 | 22.4 |  | 61 | 24.8 |  | 29 | 22.0 |  | 18 | 20.2 |  |  |
| pN |  |  | |  |  |  |  |  |  |  |  |  |  |  |  |  |  |  | < 0.001 |  |
| N0 | 1111 | 61.3 | |  | 1111 | 99.9 |  | 0 | 0 |  | 0 | 0 |  | 0 | 0 |  | 0 | 0 |  |  |
| N1a | 212 | 11.7 | |  | 0 | 0 |  | 148 | 63.8 |  | 53 | 21.5 |  | 9 | 6.8 |  | 2 | 2.3 |  |  |
| N1b | 250 | 13.8 | |  | 1 | 0.1 |  | 77 | 33.2 |  | 114 | 46.3 |  | 31 | 23.5 |  | 27 | 30.3 |  |  |
| N2a | 145 | 8.0 | |  | 0 | 0 |  | 6 | 2.6 |  | 53 | 21.5 |  | 59 | 44.7 |  | 27 | 30.3 |  |  |
| N2b | 93 | 5.1 | |  | 0 | 0 |  | 1 | 0.4 |  | 26 | 10.6 |  | 33 | 25.0 |  | 33 | 37.1 |  |  |
| EN | 10.46±6.42 | | |  | 9.97±6.21 | |  | 14.25±6.38 | |  | 10.95±6.67 | |  | 9.30±5.80 | |  | 7.09±4.94 | | < 0.001 |  |
| PN | 1.30±2.46 | | |  | 0 | |  | 1.54±0.90 | |  | 3.19±2.15 | |  | 5.06±3.10 | |  | 6.20±4.23 | | < 0.001 |  |
| NN | 9.16±6.26 | | |  | 9.97±6.21 | |  | 12.70±5.78 | |  | 7.76±4.79 | |  | 4.24±2.91 | |  | 0.89±1.16 | | < 0.001 |  |
| M |  |  | |  |  |  |  |  |  |  |  |  |  |  |  |  |  |  | < 0.001 |  |
| M0 | 1678 | 92.7 | |  | 1084 | 97.5 |  | 208 | 89.7 |  | 208 | 84.6 |  | 100 | 75.8 |  | 78 | 87.6 |  |  |
| M1 | 133 | 7.3 | |  | 28 | 2.5 |  | 24 | 10.3 |  | 38 | 15.4 |  | 32 | 24.2 |  | 11 | 12.4 |  |  |
| OS | 64.2% | | |  | 75.2% | |  | 66.1% | |  | 48.0% | |  | 34.0% | |  | 15.0% | | < 0.001 |  |

EN：examined nodes; PN: positive nodes; NN: negative nodes; OS: overall survival

**Supplementary Table 2. Multivariate analysis of pN and clinicopathologic characteristics with overall survival (OS) for colorectal cancer patients in international multicenter cohort.**

| **Characteristics** | HR | **95% CI** | | *p* |
| --- | --- | --- | --- | --- |
|  |  | Lower | Upper |  |
| **Sex** |  |  |  |  |
| Male vs Female* | 0.98 | 0.83 | 1.16 | 0.843 |
| **Age level** |  |  |  |  |
| ≥60 vs <60* | 1.73 | 1.41 | 2.11 | <0.001 |
| **Location** |  |  |  |  |
| Right side | Reference |  |  |  |
| Left side | 0.89 | 0.73 | 1.09 | 0.255 |
| Rectum | 1.30 | 1.05 | 1.61 | 0.015 |
| **Grade** |  |  |  |  |
| High grade vs Low grade* | 1.34 | 1.12 | 1.60 | 0.001 |
| **Histology** |  |  |  |  |
| Adenocarcinoma | Reference |  |  |  |
| Mucinous adenocarcinoma | 0.78 | 0.57 | 1.07 | 0.121 |
| Signet ring cell carcinoma | 5.19 | 1.91 | 14.12 | 0.001 |
| **T** |  |  |  |  |
| T1 | Reference |  |  |  |
| T2 | 1.82 | 0.80 | 4.14 | 0.151 |
| T3 | 3.24 | 1.51 | 6.99 | 0.003 |
| T4 | 3.44 | 1.56 | 7.60 | 0.002 |
| **N** |  |  |  |  |
| N0 | Reference |  |  |  |
| N1 | 1.89 | 1.56 | 2.29 | <0.001 |
| N2 | 2.93 | 2.32 | 3.70 | <0.001 |
| **M** |  |  |  |  |
| M1 vs M0* | 2.42 | 1.87 | 3.13 | <0.001 |
| **Size level** |  |  |  |  |
| ≤2 | Reference |  |  |  |
| ≤3 | 1.33 | 0.77 | 2.30 | 0.305 |
| ≤5 | 1.15 | 0.69 | 1.91 | 0.594 |
| >5 | 1.63 | 0.97 | 2.75 | 0.064 |

HR=hazard ratio; CI=confidence interval; *Reference category

**Supplementary Table 3. Multivariate analysis of Lymph Node Radio (LNR) and clinicopathologic characteristics for overall survival (OS) of colorectal cancer patients in international multicenter cohort.**

| **Characteristics** | HR | **95% CI** | | *p* |
| --- | --- | --- | --- | --- |
|  |  | Lower | Upper |  |
| **Sex** |  |  |  |  |
| Male vs Female* | 0.96 | 0.81 | 1.13 | 0.616 |
| **Age level** |  |  |  |  |
| ≥60 vs <60* | 1.73 | 1.41 | 2.11 | <0.001 |
| **Location** |  |  |  |  |
| Right side | Reference |  |  |  |
| Left side | 0.93 | 0.76 | 1.13 | 0.470 |
| Rectum | 1.18 | 0.95 | 1.47 | 0.127 |
| **Grade** |  |  |  |  |
| High grade vs Low grade* | 1.29 | 1.08 | 1.53 | 0.005 |
| **Histology** |  |  |  |  |
| Adenocarcinoma | Reference |  |  |  |
| Mucinous adenocarcinoma | 0.74 | 0.54 | 1.01 | 0.058 |
| Signet ring cell carcinoma | 3.04 | 1.11 | 8.32 | 0.031 |
| **T** |  |  |  |  |
| T1 | Reference |  |  |  |
| T2 | 1.89 | 0.83 | 4.30 | 0.128 |
| T3 | 3.37 | 1.56 | 7.25 | 0.002 |
| T4 | 3.77 | 1.71 | 8.31 | 0.001 |
| **LNR_classification** |  |  |  |  |
| LNR0 | Reference |  |  |  |
| LNR1 | 1.20 | 1.11 | 1.30 | 0.004 |
| LNR2 | 1.86 | 1.48 | 2.34 | <0.001 |
| LNR3 | 2.78 | 2.14 | 3.61 | <0.001 |
| LNR4 | 6.95 | 5.29 | 9.12 | <0.001 |
| **M** |  |  |  |  |
| M1 vs M0* | 2.73 | 2.12 | 3.52 | <0.001 |
| **Size level** |  |  |  |  |
| ≤2 | Reference |  |  |  |
| ≤3 | 1.40 | 0.81 | 2.42 | 0.223 |
| ≤5 | 1.09 | 0.65 | 1.83 | 0.750 |
| >5 | 1.73 | 1.03 | 2.92 | 0.038 |

HR=hazard ratio; CI=confidence interval; *Reference category

## Supplementary Figures:


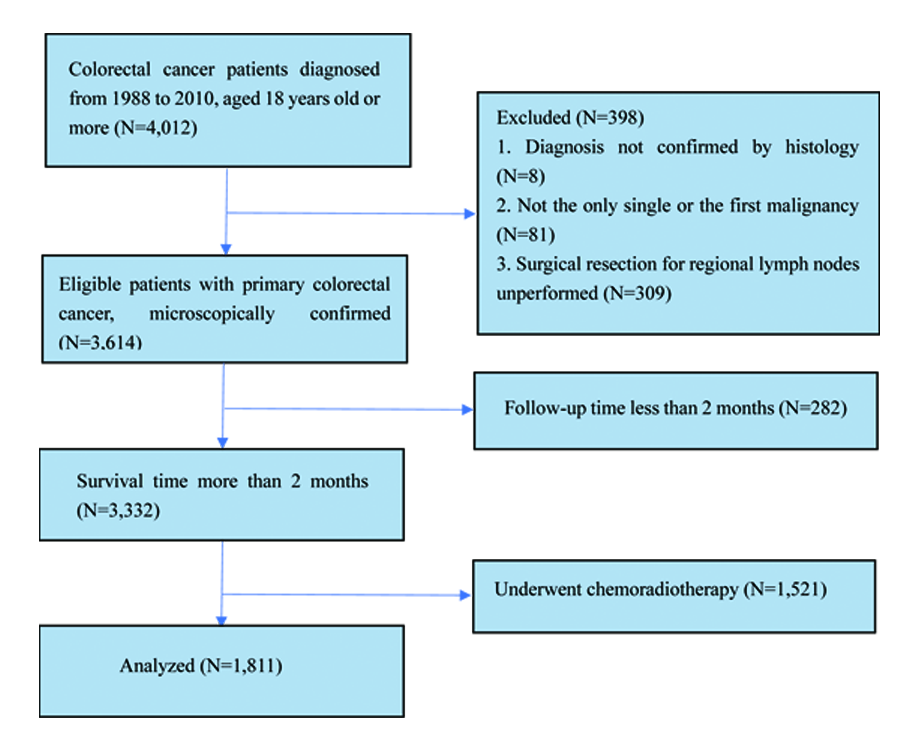


**Supplementary Figure 1.** Flow chart of patients’ cohort definition from International multicentre cohort


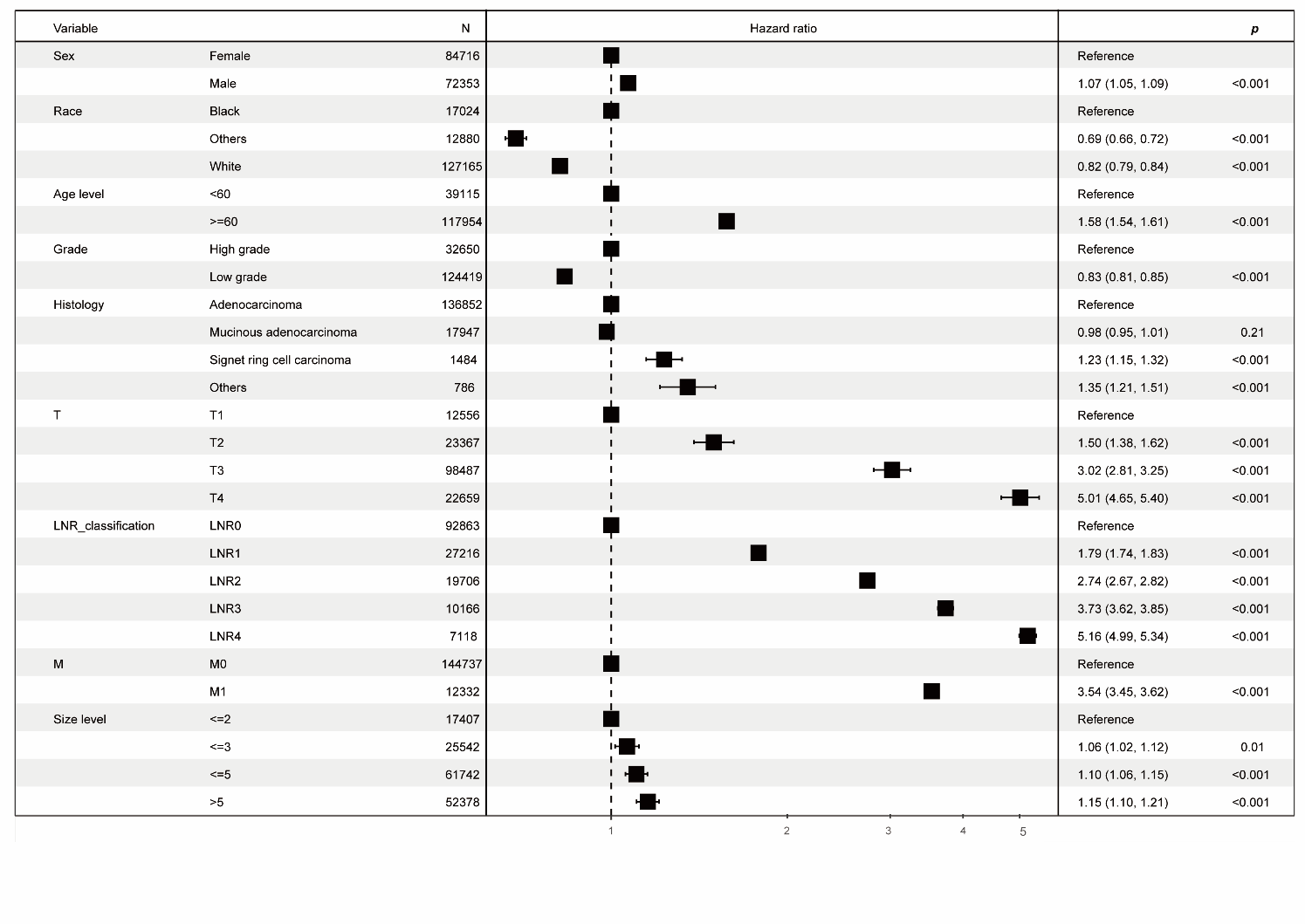


**Supplementary Figure 2**. Multivariate analysis of Lymph Node radio (LNR) and clinicopathologic characteristics for cancer-specific survival (CSS) of colon rectal patients in SEER database.


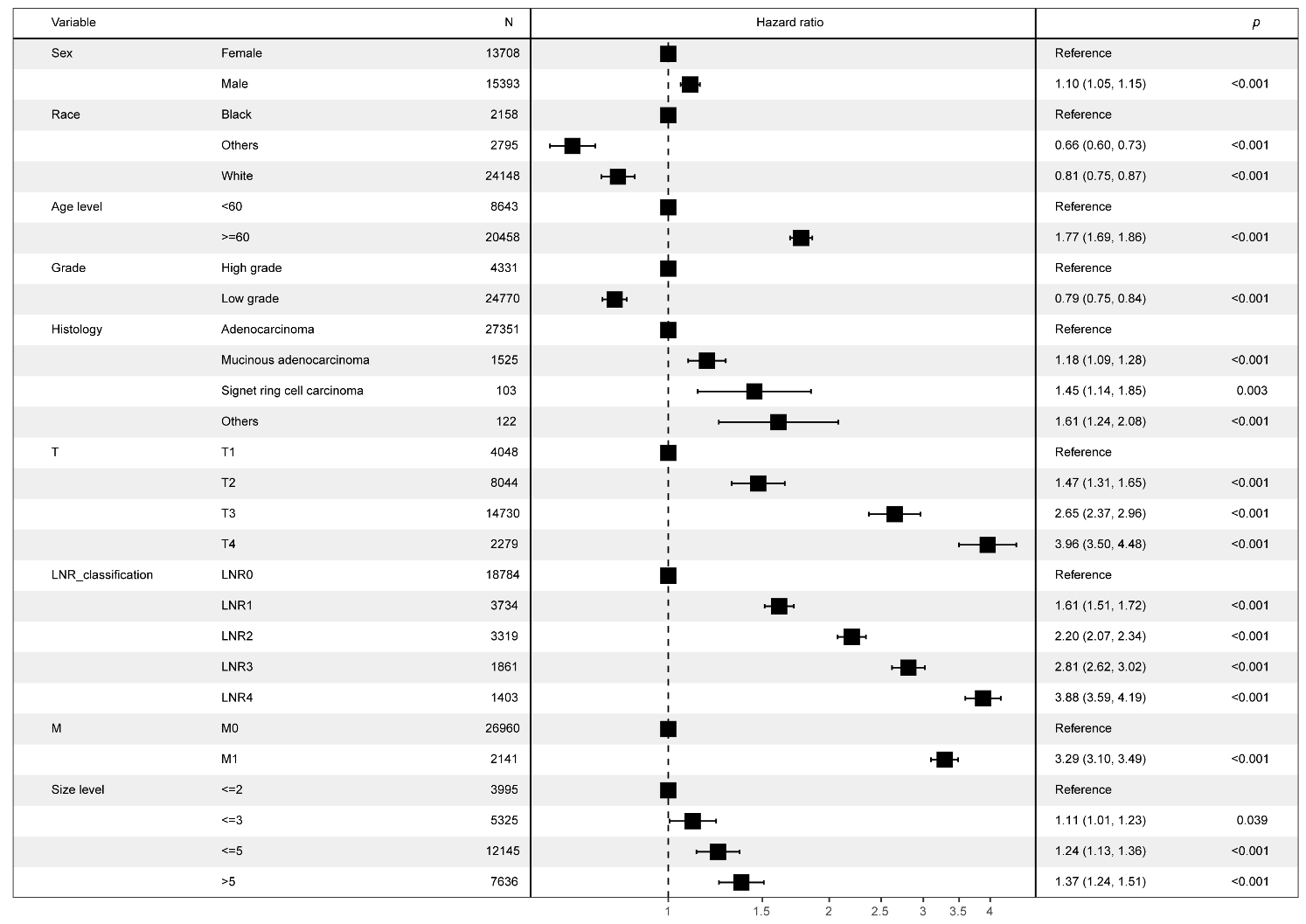


**Supplementary Figure 3**. Multivariate analysis of Lymph Node radio (LNR) and clinicopathologic characteristics for cancer-specific survival (CSS) of rectal cancer patients in SEER database.


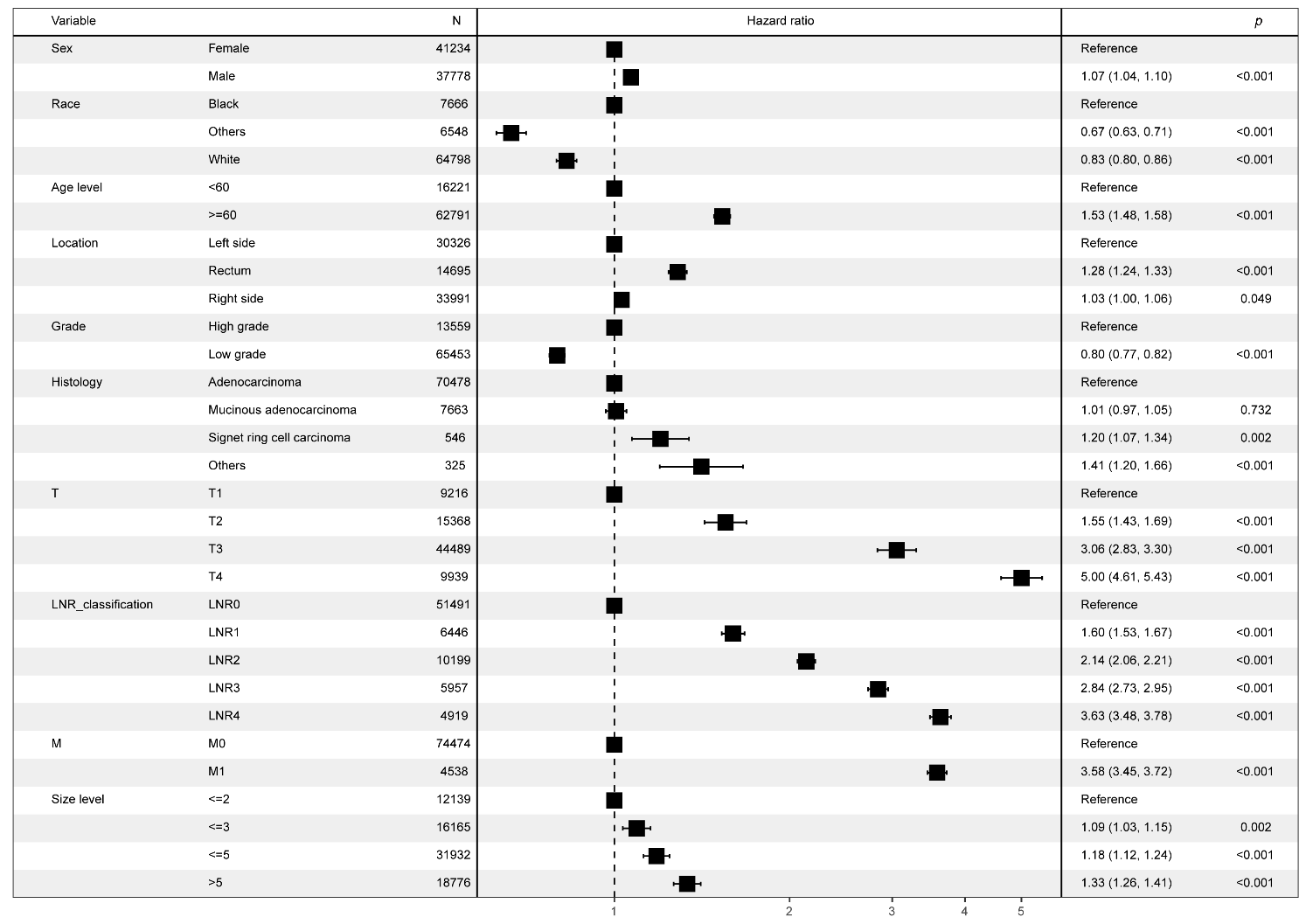


**Supplementary Figure 4**. Multivariate analysis of Lymph Node radio (LNR) and clinicopathologic characteristics for cancer-specific survival (CSS) of colorectal cancer patients when <12 nodes were removed in SEER database.


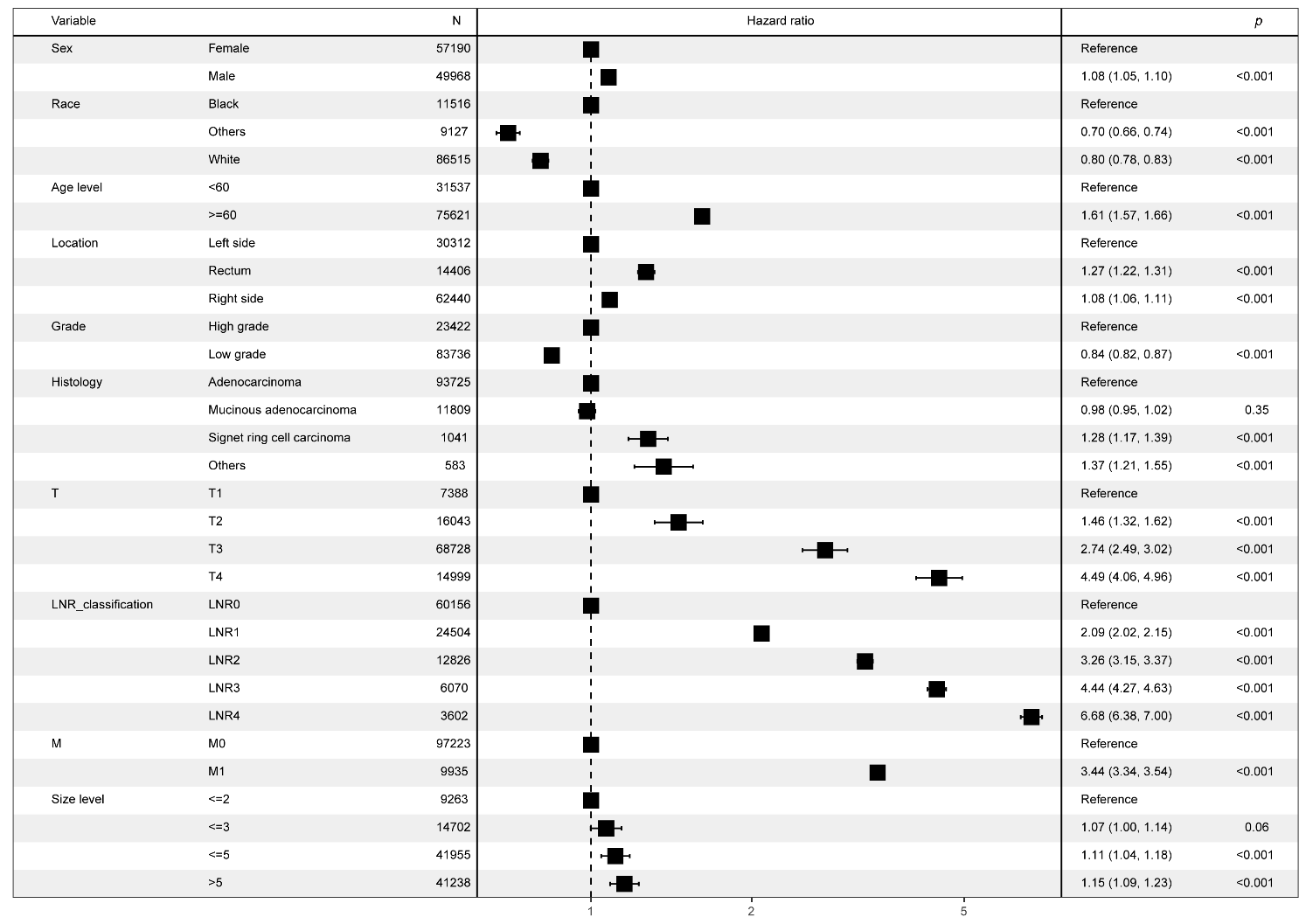


**Supplementary Figure 5**. Multivariate analysis of Lymph Node radio (LNR) and clinicopathologic characteristics for cancer-specific survival (CSS) of colorectal cancer patients when ≥12 nodes were removed in SEER database.
